# Supplementary material for: Transgenesis enables mapping of segmental ganglia in the leech Helobdella austinensis
Source: J Exp Biol. 2024 Jul 24;227(14):jeb247419. doi: 10.1242/jeb.247419 (PMC11418187; doi:10.1242/jeb.247419)
Supplement: Supplementary information [file jexbio-227-247419-s1.pdf]

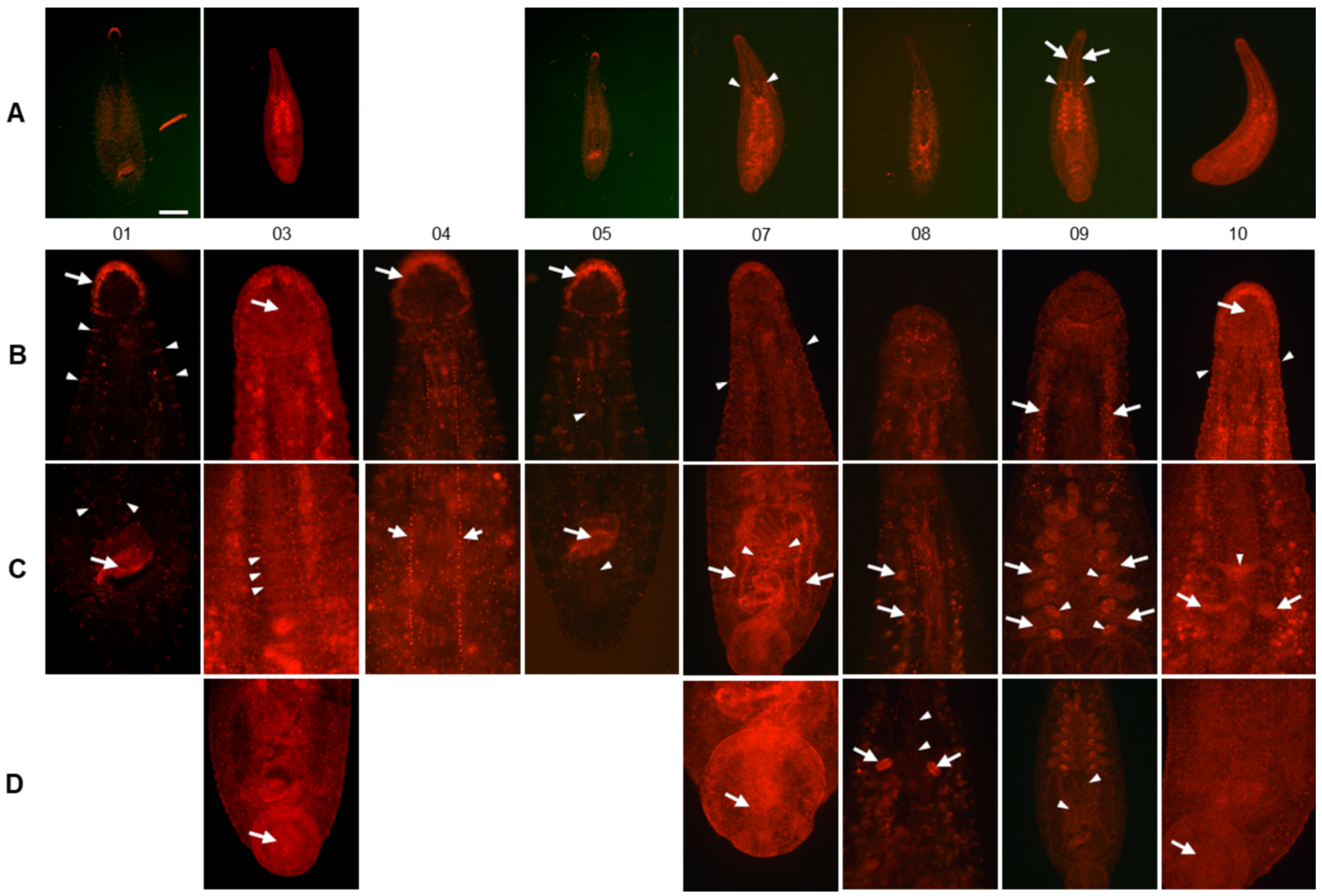

Fig. S1. Variability in transgene expression in a breeding population of transgenic *Helobdella austinensis*. Fluorescence images showing all (row A) or selected regions (rows B, C and D) of ten individuals drawn from a breeding colony of animals expressing *ef1a-h2b:mCh*. The colony was initiated with a cohort of mCherry-positive F0 individuals and thus is expected to represent a range of transgene locations and copy number. Variability among individuals was observed in terms of overall brightness (cf A1, A5 vs A3, A7, A10), and in the nuclei of specific cell types or tissues including: rim of the anterior sucker (arrows in B1, B4, B5), the overall anterior sucker (arrows in B3, B10), posterior sucker (arrows in D3, D7, D10), oblique muscles (arrows in C4), crop seca (arrows in C7, C9), intestine (arrowheads in C7, D9), anterior rectum (arrows in C1, C5), segmental ganglia (arrowheads in B5, C5, D8), atrium of the male reproductive system (arrowhead in C10), ovaries (arrows in C10, D8), testis (arrowheads in C9), central annuli of the body wall segments (arrowheads in B1), all annuli (arrows in B7, B10), salivary glands (arrowheads in A7, A9), patches of uniformly sized nuclei (arrows C8), sub-annular belts (arrowheads in C3), previously undescribed triplets of cells (arrowheads C1), previously undescribed bilateral anterior rows of cells (arrows in A9, A10, B9). Anterior is up in all images; scale bar, 1 mm in row A.

Journal of Experimental Biology • Supplementary information

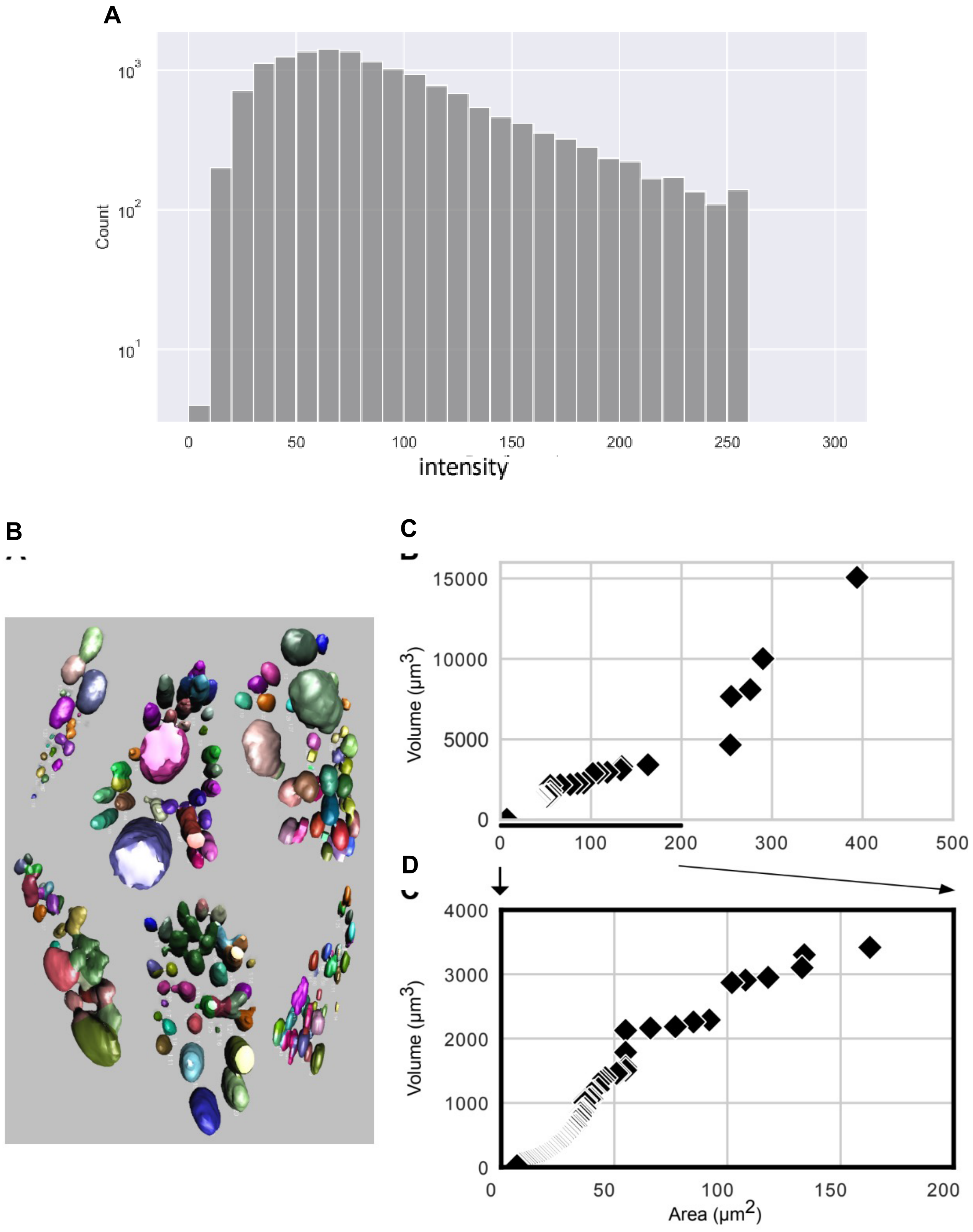

Fig. S2. Correlation of volume and projected area of neuronal nuclei. A. Intensity histogram (y-axis in log scale) shows the distribution of intensities (average intensity of all the pixels in the object) for all mCherry-labeled neuronal nuclei from all three specimens used in this study. **B.** 3D representation of all the neuronal nuclei from ganglion M08 in L3. **C.** Area of M08 nuclei vs volume of nuclei in A. **D.** As in B, for a narrower volume range.

Journal of Experimental Biology • Supplementary information

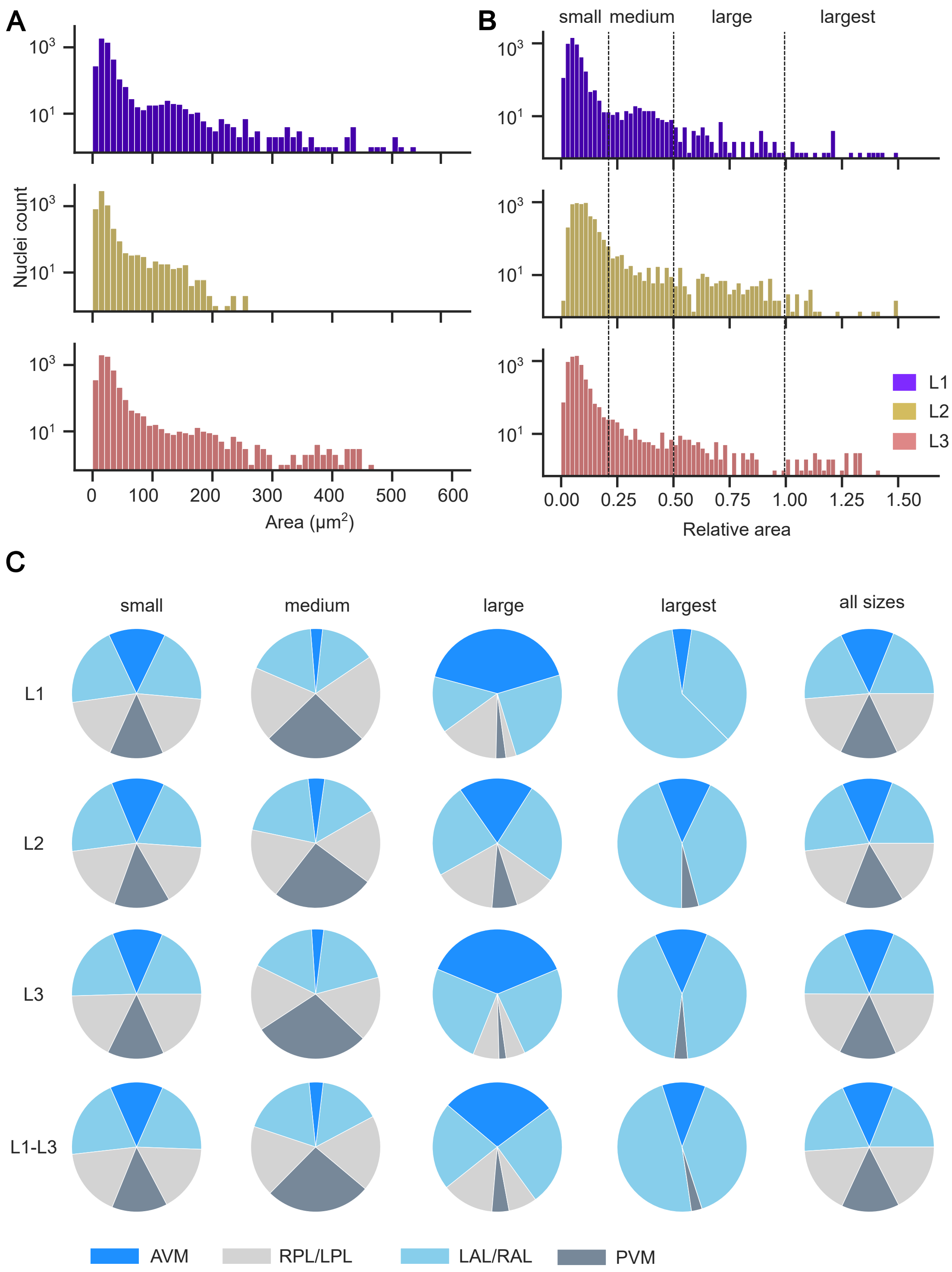

Fig. S3. Analysis of nuclear size distribution in L1, L2 and L3. A. Histogram of neuronal nucleus size distribution in each of the three leeches analyzed. **B.** As in A but for the relative area, calculated as a proportion of the mean 1% largest area in each animal. **C.** Distribution of small, medium, large and largest nuclei among different packets for each leech and for the three leeches together (as in Fig. 7C); and distribution of the number of neurons per packet for each leech and for the three leeches together (as in Fig. 7B).

Journal of Experimental Biology • Supplementary information

Table S1. Primers for cloning cDNA and gDNA fragments used in this study

**Targeted product**	**Primer pairs**
Hau-elav1 cDNA fragment (Hro 187316 ortholog)	Fw: GGGTTCGTTGGAGGAAGATAA<br>Re: TGCACTAGATGGTGCTTGCAA
Hau-elav2 cDNA fragment (Hro 83112 ortholog)	Fw: CTCTAGCGTTGGTGAAATCGA<br>Re: GTATTCTTCCTGCCAGCTGAT
Hau-elav3 cDNA fragment (Hro 62839 ortholog)	Fw: TGGAAGCATTGGAGAGATGGA<br>Re: CATGATAATCGGTCATGGTGA
Hau-elav4 cDNA fragment (Hro 185541 ortholog)	Fw: AGGGATGGACTCGCACCAGGA<br>Re: TACTGTAAGGTAGGAATGGTC
Hau-elav4 (-3,549::+3) genomic DNA fragment	Fw: AGATTGAACGGACAAGCGTTAGACGT<br>Re: CATCCCTATTGCTGTCTGGACGGT
Hau-cif7 (-4254::+24) genomic DNA fragment	Fw: GCAGGCAACGCTAAGCTTTCTGA<br>Re: GCCGCTCGAACGATAGTCAGACAT

Table S2. Primers for building pMiEF1P-derived constructs

**Resulting construct**	**Primer pairs**
pMi{Htr-efla-ngfp}	Fw: GATCATCGATATGGCTCCAAAGAAGAAGCGTAAGGTA<br>Re: GATCTCGAGCTACTTGTACAGCTCGTCCATGCCG
pMi{Htr-efla-h2b:gfp} and pMi{Htr-efla-h2b:mcherry}	Fw: GATCATCGATATGCCACCAAAGCCTGCCAGC<br>Re: GATCTCGAGCTACTTGTACAGCTCGTCCATGCCG

Note: Underline marks inserted restriction sites for cloning into pMiEF1P-SV40.

Journal of Experimental Biology • Supplementary information

Table S3. Primers for tissue-specific reporters.

Targeted product	Primer pairs
pMi{Hau-elav4-ngfp}	Elav4P (Template: *Hau-elav4* (-3,549::+3) genomic DNA fragment)<br>Fw: GATCTGCAAATAATGCTGCACGCGGATTAATCACTCACTTTCCCT<br>Re: TATTGCTGTCTGGACGGTTTTTTTTTTGGT<br><br>nGFP-SV40 (Template: pCS2+nGFP)<br>Fw: CCGTCCAGACAGCAATAGGGCCACCATGGCTCCAAAGAAGAAGCGT<br>Re: AGAATTGTGTAACGTCCCGCGAATTAAAAAACCTCCCACACCTCC
pMi{Hau-elav4-h2b:mcherry}	Elav4P (Template: *Hau-elav4* (-3,549::+3) genomic DNA fragment)<br>Fw: GATCTGCAAATAATGCTGCACGCGGATTAATCACTCACTTTCCCT<br>Re: TATTGCTGTCTGGACGGTTTTTTTTTTGGT<br><br>H2B-mCherry-SV40 (Template: pCS107 H2B-mCherry)<br>Fw: CCGTCCAGACAGCAATAGGGCCACCATGCCACCAAAGCCTGCCA<br>Re: AGAATTGTGTAACGTCCCGCGAATTAAAAAACCTCCCACACCTCC
pMi{Hau-cif7-ngfp}	Cif7P (Template: *Hau-cif7* (-4254::+24) genomic DNA fragment)<br>Fw: GATCTGCAAATAATGCTGCAGGCAACGCTAAGCTTT<br>Re: CGTCTGTAAATATTTTATTATTTTTCAAATAAATCGA<br><br>nGFP-SV40 (Template: pCS2+nGFP)<br>Fw: TAAAATATTTACAGACGCCACCATGGCTCCAAAGAAGAAGCGT<br>Re: AGAATTGTGTAACGTCCCGCGAATTAAAAAACCTCCCACACCTCC

Note: Underline marks overlapping sequences required for DNA assembly reaction.

Table S4. Primers for genotyping *Hau-elav4* reporter transgenic lines

Forward primer	TGAGTTTGGACAAACCACAACTA
Reverse primer	CCCCATCTTACATTTCTTTCCGT

Dataset 1. Raw data from anatomical analysis

Available for download at
https://journals.biologists.com/jeb/article-lookup/doi/10.1242/jeb.247419#supplementary-data

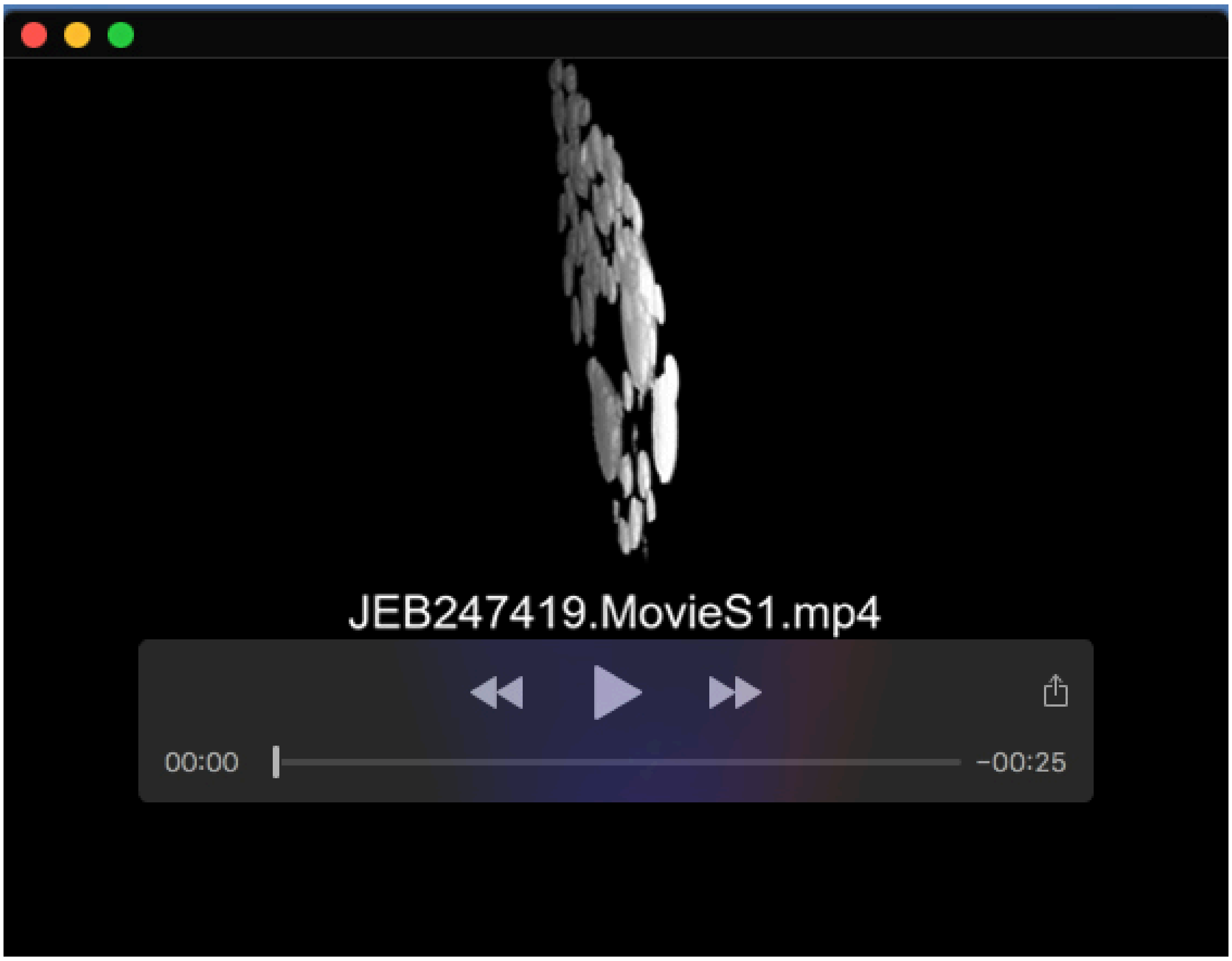

Movie 1. 180-degree rotated rendered 3D images revealed nuclei hidden behind large neurons on the dorsal side of the ganglion

Journal of Experimental Biology • Supplementary information
